# Will Obstructive Sleep Apnea and Apnea/Hypopnea Index Be Corrected Following Alveolar Cleft Reconstruction?

**DOI:** 10.29252/wjps.9.2.146

**Published:** 2020-05

**Authors:** Sahand Samieirad, Alireza Khoshsirat, Fariba Rezaeetalab, Vajiheh Mianbandi, Elahe Tohidi, Majid Eshghpour

**Affiliations:** 1 Oral and Maxillofacial Diseases Research Center, Mashhad University of Medical Sciences, Mashhad, Iran;; 2 Oral and Maxillofacial Surgery Department, Dental School, Mashhad University of Medical Sciences, Mashhad, Iran;; 3 Lung Disease Research Center, Mashhad University of Medical Sciences, Mashhad, Iran;; 4 Student Research Committee, Dental School, Mashhad University of Medical Sciences, Mashhad, Iran;; 5 Oral and Maxillofacial Radiology Department, Dental Research Center, Mashhad University of Medical Sciences, Mashhad, Iran

**Keywords:** Obstructive sleep apnea, Alveolar cleft, Apnea, Hypopnea

## Abstract

**BACKGROUND:**

Obstructive sleep apnea is a disorder of repetitive complete or partial airway obstruction during sleep. The aim of this study was to assess the impact of alveolar cleft reconstruction on the obstructive sleep apnea (OSA) condition and apnea/hypopnea index (AHI).

**METHODS:**

In a double-blinded prospective quasi-experimental study, all healthy systemic children (n=30 female cleft patients) with unilateral alveolar cleft defects within the age range of 8-14 years and BMI less than 30 kg/m^2^ who admitted for alveolar cleft repair were enrolled. OSA monitoring was performed one week before surgery, and 3 months postoperatively by Apnea Link device. Sleep apnea indices such as AHI, respiratory disturbance index (RDI), oxygen desaturation index (ODI) and oxyhemoglobin saturation (SpO2) as well as pulse rate (PR) and respiratory rate (RR) were the variables.

**RESULTS:**

The patients’ mean age was 11.0±1.4 years, and BMI average was 21.48±4.4 kg/m^2^. Mean AHI was 21.6±5.0 events/hour, preoperatively; which decreased significantly and reached 4.4±2.5 events/hour after alveolar cleft reconstruction surgery (*p*=0.005). Moreover, the other OSA variables (SpO2, RDI, and ODI), as well as vital signs (PR, and RR) improved postoperatively (*p*=0.005). In other words, the preoperative moderate OSA status relieved after alveolar cleft repair and reconstruction.

**CONCLUSION:**

Our study showed that the OSA and AHI ameliorated after bone graft surgery in alveolar cleft repair. More clinical trials including larger sample sizes may be required for relevancy.

## INTRODUCTION

Obstructive sleep apnea is a disorder of repetitive complete or partial airway obstruction during sleep.^1^^-^^3^ Symptomatic obstructive sleep apnea is a common, underdiagnosed condition that occurs in 1.2-5.8% of the pediatric population.^1^^,^^4^^,^^5^ Risk factors for obstructive sleep apnea (OSA) include adenotonsillar hypertrophy, obesity, craniofacial anomalies, and neuromuscular disorders.^1^^,^^2^^,^^4^^-^^6^ Accurate diagnosis is required not only to ensure proper treatment of sleep apnea but also to prevent the possible complications and comorbidities, such as systemic and pulmonary hypertension, neurocognitive impairment, and behavioral problems, as well as excessive daytime sleepiness and concentration difficulties.^2^^,^^6^^,^^7^

Cleft palate is one of the most frequently occurring congenital malformations with an incidence of 0.69-2.51 per 1000 births.^3^^,^^6^^,^^8^ The recent studies estimated the risk of OSA to be between 22% and 65% in infants and children with cleft lip/palate (CL/P).^2^^,^^3^^,^^6^^,^^8^^,^^9^ Children with craniofacial deformities, including CL/P and alveolar cleft, have been identified to be at the high risk of OSA due to nasopharyngeal and oropharyngeal anatomical abnormalities which may be congenital or acquired as a result of surgical intervention.^6^^,^^9^^-^^11^

The review of literature depicted that the palatoplasty, pharyngoplasty, and pharyngeal flap, usually performed for oral rehabilitation in maxillofacial cleft patients, increase the risk of OSA.^2^^,^^3^^,^^6^^,^^9^^,^^11^^-^^14^ To the best of our knowledge, no previous study has evaluated obstructive sleep apnea recordings before and after surgery in a pediatric group of maxillary alveolar cleft patients undergoing cleft defect repair and bone grafting reconstruction procedure.^2^^,^^3^^,^^7^^,^^9^^-^^14^ The purpose of this study was to assess the impact of alveolar cleft repair and reconstruction using bone graft on the OSA condition and apnea/hypopnea index (AHI).

## MATERIALS AND METHODS

A double-blind prospective quasi-experimental trial was carried out. Its protocol was approved by the Ethics and Research Committee of Mashhad University of Medical Sciences (IR.MUMS.DENTISTRY.REC.1397.013) and it was regis­tered under the code of IRCT20150613022697N3. Guidelines of the Declaration of Helsinki were followed in this research. Informed written consent was collected from all participants’ parents prior to enrollment.

All the healthy children (ASA I and II) who had unilateral alveolar cleft defects within the age range of 8 to 14, and with BMI less than 30 kg/m^2^ (non-obese) were included in this study. These cases were candidates for alveolar cleft repair and reconstruction with iliac bone graft in the Maxillofacial Surgery Department from February to December 2018. All subjects had a history of successful cheilorrhaphy and palatoplasty operations. However, subjects with bilateral alveolar clefts and large adenoid tonsils, as well as cases with the history of pharyngoplasty or pharyngeal flaps were excluded from the present study to eliminate the additional confounding factors.

They did not have respiratory diseases such as rhinitis, asthma, and bronchitis. In addition, the patients with serious medical conditions (ASA³III), history of cardiovascular, pulmonary and renal diseases, as well as those who refused the follow-up checkups were excluded. The cases were examined pre-operatively by a single respiratory physician (who was expert in sleep apnea studies), to rule out respiratory and cardiovascular diseases. The obstructive sleep apnea was confirmed for all of our cases regarding the clinical symptoms.

All operations for repair and reconstruction of alveolar cleft defects were carried out by a single surgeon with the same protocol using local buccal, palatal, and nasal flaps. The authors performed the dissection up to the piriform rim in order to augment this area and provide the support for the nasal base. Then, the iliac corticocancellous bone was placed into the defect to reconstruct the defect. The portable home monitoring device we used was Apnea Link™ (Res Med Corporation, Poway, California, USA). Subjects’ parents were instructed how to use the Apnea Link device by our staff at the sleep center. 

Subjects also agreed to use the Apnea Link device at home for 1 night, both pre- and post-operatively. All participants were monitored for obstructive sleep apnea one week before surgery, and 3 months after surgery. It should be noted that the person responsible for data collection and principle investigation (the pulmonologist who was expert in sleep apnea) and the data analyzer were blind towards the study design (double-blind study). The study intervention was the alveolar cleft reconstruction using iliac bone graft. Sleep apnea indices such as AHI, respiratory disturbance index (RDI), oxygen desaturation index (ODI) and oxyhemoglobin saturation (SpO2) were the study primary outcome variables. 

**Fig. 1:**
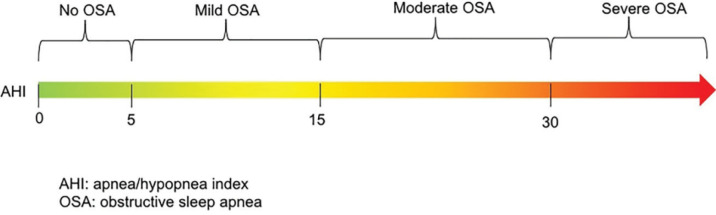
The classification of OSA severity based on AHI

The AHI reported the average events of apneas and hypopneas per hour during sleep. This index determines OSA disease severity. Classification of OSA severity based on AHI value was presented in Figure 1. Apnea was defined as a drop in airflow of at least 90% lasting at least 10 seconds, and hypopnea is defined as an event lasting more than 10 seconds with a greater than 30% reduction in airflow accompanied by either an arousal or greater than 3% oxygen desaturation from the pre-event baseline.^1^^,^^15^^-^^18^

The RDI represents the number of apneas, hypopneas, and respiratory events related to arousals (RERAs) per hour. ODI is defined as the average number of oxygen desaturations per hour during sleep.^1^^,^^15^^-^^18^ The peripheral capillary oxygen saturation (SpO_2_) is the percentage of oxygenated hemoglobin versus the total amount of hemoglobin in blood.^1^^,^^15^^-^^18^ Data were analyzed using SPSS software for Windows (Version 16, SPSS Inc., Chicago, IL, USA). We employed paired t-test, Wilcoxon, and Spearman tests. *p* value<0.05 was considered statistically significant.

## RESULTS

The present study included 30 patients sustaining unilateral alveolar cleft with the average age of 11.0±1.4 and age range of 9 to 14 years. All cases were selected from female patients to reduce confounding sex factors. Mean body mass index (BMI) of patients was 21.48±4.4 kg/m^2^. As demonstrated in Table 1, all the variables associated with sleep apnea decreased and improved after bone graft surgery in alveolar cleft patients, compared to the preoperative condition. All variables alterations were statistically significant (*p*=0.005). 

**Table 1:** The changes of obstructive sleep apnea indexes after alveolar cleft reconstruction (n=30)

**Variable**	**Normal range**	**Time of measurement**	**Mean**	**Standard deviation**	**Min**	**Max**	***p*** ** value**
AHI	<5	Pre-operatively	21.6	5.0	15.8	28.4	^*^0.005
		Post-operatively	4.4	2.5	2.7	11.2	
RDI	<5	Pre-operatively	25.9	4.8	18.2	30.4	^*^0.005
		Post-operatively	6.0	3.2	3.2	14.8	
ODI	<5	Pre-operatively	24.4	3.3	18.7	27.5	^*^0.005
		Post-operatively	4.3	2.9	1.8	11.6	
SpO2 (%)	>90	Pre-operatively	92.6	1.35	91	95	^*^0.005
		Post-operatively	96.3	0.67	95	97	
Mean RR	12-20	Pre-operatively	17.3	3.2	12.99	22.70	^**^0.001
		Post-operatively	13.1	1.2	11.2	15.0	
Mean PR	60-100	Pre-operatively	97.1	4.0	90	103	^*^0.005
		Post-operatively	78.4	4.6	67	83	

*: Wilcoxon test , **: Paired t-test. AHI: Apnea/Hypopnea Index, RDI: Respiratory disturbance index, ODI: Oxygen desaturation Index, PR: Pulse rate, RR: Respiratory rate. SpO_2_: The peripheral capillary oxygen saturation

Mean AHI was 21.6±5.0 events per hour preoperatively and decreased significantly and reached 4.4±2.5 events per hour after alveolar cleft repair surgery (*p*=0.005). In other words, the preoperative moderate OSA was ameliorated after bone graft surgery and alveolar cleft repair. It is noteworthy that SpO_2_ was 92.6%±1.35 before the surgery and increased 3.995% and reached 96.3±0.67, postoperatively (*p*=0.005). Preoperatively, SpO_2_% was recorded to be within the normal range, which means that none of the participants suffered respiratory difficulties (Table 1).

**Fig. 2:**
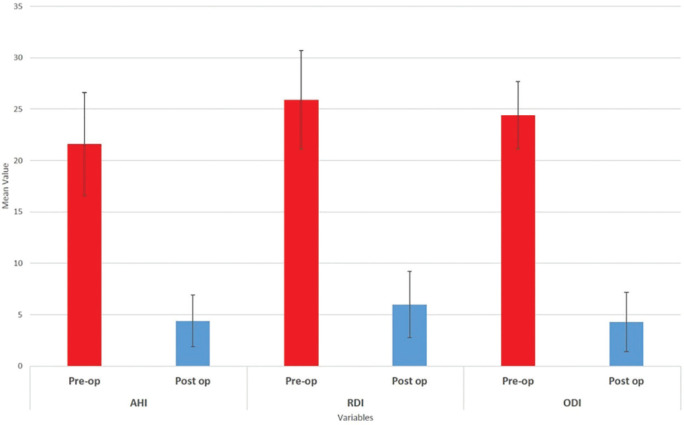
The comparison of obstructive sleep apnea indices before and after the alveolar cleft repair. AHI: Apnea/Hypopnea Index, RDI: Respiratory disturbance index, ODI: Oxygen desaturation Index

Actually, the most important OSA variables were corrected and improved after alveolar cleft reconstruction. This fact was better illustrated in Figure 2. BMI indicated a significant positive correlation with the indices associated with obstructive sleep apnea (AHI, RDI, and ODI), preoperatively. An increase in BMI led to an increase in the mentioned variables, resulting in a higher risk of OSA and vice versa. Moreover, these relations were statistically significant (Table 2) 

**Table 2:** Spearman correlation coefficient between BMI and age parameters with the sleep apnea variables (n=30)

	**Variable**	**AHI**	**RDI**	**ODI**
BMI	Spearman Correlation Coefficient	0.831	0.663	0.810
	*p* value	0.003	0.037	0.005
Age	Spearman Correlation Coefficient	0.569	0.241	0.361
	*p* value	0.086	0.503	0.305

AHI: Apnea/Hypopnea Index, RDI: Respiratory disturbance index, ODI: Oxygen desaturation Index, BMI: Body mass index

The results in the Table 2 depicted that there was a positive correlation between patients’ age and the variables associated with obstructive sleep apnea, preoperatively. Along with aging, the amount of these indices (AHI, RDI, and ODI) increased and led to a higher risk of obstructive sleep apnea and vice versa. However, these relations were not statistically significant.

## DISCUSSION

In recent years, studies have focused on the effects of maxillofacial surgeries on the airway, respiration and OSA.^1^^,^^4^^,^^8^^,^^9^^,^^19^ OSA is characterized by pauses in breathing during sleep due to collapse or blockage of the upper airways. Repeated cessation (apnea) or reduction (hypopnea) of respiration may lead to excessive daytime sleepiness, headache, neurocognitive problems and depression as well as pulmonary hypertension, diabetes, cardiovascular and renal diseases.^1^^,^^2^^,^^7^^,^^15^^,^^16^^,^^19^^,^^20^

Airway obstruction and OSA in the cleft lip and palate population have long been recognized as a complication of the surgeries provided to restore speech.^6^^,^^9^^-^^11^ Our study findings showed that the preoperative moderate OSA was relieved after alveolar cleft repair surgery and reconstruction with bone graft. In children with CP/L, the associated facial features including midface hypoplasia and a retrognathic maxilla may disturb the upper airway dimensions that persist even after surgical correction.^2^^,^^6^^,^^7^^,^^11^

Regarding the literature, most of the surgeries associated with palatoplasty, sphincter pharyngoplasty, and pharyngeal flaps in cleft lip/palate children worsen the obstructive sleep apnea condition, as these procedures often decrease the cross-sectional area of the airway.^2^^,^^3^^,^^8^^,^^11^^-^^13^ Despite the adverse effects of OSA on health, little is known about the changes of OSA and AHI following the surgical intervention of maxillary alveolar cleft repair.^2^^,^^11^ Multiple studies have demonstrated that OSA questionnaires, history and physical examinations are not reliable enough in diagnosing OSA.^2^^,^^3^^,^^6^^,^^7^

Polysomnography (PSG) is a useful tool and the gold standard for detecting sleep disorders.^1^^,^^3^^,^^5^^,^^10^^,^^15^^,^^18^ Unfortunately, PSG studies are labor intensive, time-consuming, and expensive to perform.^1^^,^^3^^,^^5^^,^^10^^,^^15^^,^^18^ These issues have led to the development of ambulatory sleep monitors and portable recording devices such as Apnea Link polygraph device to assess OSA in a variety of settings (e.g. patient’s home or hospital).^17^^,^^18^ The Apnea Link™ is an ambulatory sleep monitor that can detect OSA with acceptable reliability, according to recent articles.^5^^,^^17^^,^^18^

When compared to PSG, the Apnea Link™ had a sensitivity of 97.7% and a specificity of 93.9%, with different AHI cutoffs from 5 to 30 events per hour.^17^^,^^18^ Given the costs of in-laboratory PSG, ambulatory methods of polygraphy are desirable for diagnosis of sleep disorders.^5^^,^^17^^,^^18^ Therefore, the authors used the Apnea Link screening device to evaluate obstructive sleep apnea. The authors excluded patients with bilateral alveolar clefts and large adenoid tonsils, as well as cases with the history of pharyngoplasty or pharyngeal flaps to eliminate the confounding factors for OSA screening.^2^^,^^11^^-^^13^^,^^21^

Moreover, the arrangement of obstructive sleep apnea screening by the pulmonologist was not applicable for all patients.^1^ Considering this research inclusion and exclusion criteria, our sample size including 30 patients was suitable. It was similar to the sample size of Liao *et al.*’s study which assessed the OSA incidence after palatoplasty and pharyngeal flap surgeries.^13^ However, it was more that Eriksen *et al.*’s study which evaluated OSA and AHI in 8 patients after intraoral vertical ramus osteotomy.^1^

Only the girls with alveolar cleft were included in the present experiment. Regarding the literature, the incidence of OSA in males is about 2 times more than females.^2^^,^^3^^,^^6^^,^^7^^,^^11^ Thus, the authors omitted the boys from this study to eliminate the impact of gender on OSA results and to avoid the unintentional bias. The age range of our patients was within the golden time for secondary alveolar bone grafting (8-14 years old, depending on eruption status of permanent maxillary canine).^21^^-^^23^

The aforesaid timing is the standard approach in most cleft centers around the world.^21^^-^^23^ It should be noted that all of the maxillary alveolar cleft patients are reconstructed using bone grafts regarding our center protocol; therefore, the control group was not applicable here. Sleep apnea monitoring was performed one week before surgery, and 3 months after surgery in all of our patients. This protocol was similar to Mulgrew and Liao *et al.*’s studies, as the postoperative facial edema will be completely eliminated after 3 months in the cleft patients following bone grafting.^5^^,^^13^

In addition, the results of sleep apnea monitoring one night preoperatively might not be valid, because of the preoperative stress impact on sleep quality.^5^^,^^13^ Our study results illuminated that all variables associated with sleep apnea, especially AHI, decreased and improved significantly after bone graft surgery in alveolar cleft patients, compared to the preoperative condition (*p*=0.005). This finding can be explained by the fact that both the maxillary defect and piriform rim were augmented and reconstructed with bone grafts, which provided support for the nasomaxillary unit, consequently resulting in better respiration.^21^^-^^24^

Moreover, there are multiple advantages of reconstructing the alveolar cleft of the maxilla. The most basic outcome is to eliminate oral and nasal communication and to establish normal anatomic boundaries of the oral and nasal cavities. Unification of the cleft maxillary segments into a continuous osseous structure and establishment of intact alveolar and palatal bones to support patent anterior airway are the other primary advantages, as they can convert turbulent air flow to laminar flow.^21^^-^^24^

Regarding the improvement of OSA in our subjects, the increasing SPO_2_ percentage is logical and predictable.^1^^,^^15^^,^^16^ It should be noted that OSA amelioration and recovery decreased the patient cardiopulmonary stress which might have led to a decrease in mean pulse and respiratory rates.^1^^,^^15^^,^^16^ In addition, the observed positive correlations between AHI and patients’ age and BMI were in accordance with published papers on OSA which reported more incidence of the apnea in fat and old cases.^1^^,^^6^^,^^7^^,^^11^^,^^15^^,^^16^

Finally, since the symptoms of OSA are common in children with palatal and alveolar cleft, they are at a significant risk of OSA in early childhood which importance has been under-recognized.^3^^,^^9^^,^^14^^,^^16^ Therefore, greater care should be taken to evaluate these patients for sleep abnormalities to improve their quality of life.^11^^,^^17^ One of the present study limitations was the sample size, so we suggest recruiting a larger sample size with a higher number of alveolar cleft patients, as well as employing a control group for fu­ture studies to signify the correlation between the factors. 

The presence of OSA in children with CL/P may be influenced by several factors including age, BMI, and surgical repair of the cleft. Our study indicated that the OSA and AHI can be ameliorated after alveolar cleft repair using bone graft. Further clinical trials are required to confirm relevancy.
